# Pilot study suggests DNA methylation of the glucocorticoid receptor gene (NR3C1) is associated with MDMA-assisted therapy treatment response for severe PTSD

**DOI:** 10.3389/fpsyt.2023.959590

**Published:** 2023-02-06

**Authors:** Candace R. Lewis, Joseph Tafur, Sophie Spencer, Joseph M. Green, Charlotte Harrison, Benjamin Kelmendi, David M. Rabin, Rachel Yehuda, Berra Yazar-Klosinski, Baruch Rael Cahn

**Affiliations:** ^1^School of Life Sciences, Arizona State University, Tempe, AZ, United States; ^2^Neurogenomics Division, Translational Genomics Research Institute (TGen), Phoenix, AZ, United States; ^3^Modern Spirit, Phoenix, AZ, United States; ^4^MAPS Public Benefit Corporation, San Jose, CA, United States; ^5^Department of Psychiatry, School of Medicine, Yale University, New Haven, CT, United States; ^6^The Board of Medicine, Pittsburgh, PA, United States; ^7^Department of Psychiatry, Icahn School of Medicine at Mount Sinai, New York, NY, United States; ^8^Department of Psychiatry, James J. Peters VA Medical Center, Bronx, NY, United States; ^9^Department of Psychiatry and Behavioral Sciences, University of Southern California, Los Angeles, CA, United States; ^10^Brain and Creativity Institute, University of Southern California, Los Angeles, CA, United States

**Keywords:** PTSD, MDMA, MDMA-assisted therapy, HPA, *NR3C1*, glucocorticoid receptor, epigenetics, DNA methylation

## Abstract

**Background:**

Previous research has demonstrated that epigenetic changes in specific hypothalamic-pituitary-adrenal (HPA) genes may predict successful psychotherapy in post-traumatic stress disorder (PTSD). A recent Phase 3 clinical trial reported high efficacy of 3,4-methylenedioxymethamphetamine (MDMA)-assisted therapy for treating patients with severe PTSD compared to a therapy with placebo group (NCT03537014). This raises important questions regarding potential mechanisms of MDMA-assisted therapy. In the present study, we examined epigenetic changes in three key HPA axis genes before and after MDMA and placebo with therapy. As a pilot sub-study to the parent clinical trial, we assessed potential HPA epigenetic predictors for treatment response with genomic DNA derived from saliva (MDMA, *n* = 16; placebo, *n* = 7). Methylation levels at all 259 CpG sites annotated to three HPA genes (*CRHR1*, *FKBP5*, and *NR3C1*) were assessed in relation to treatment response as measured by the Clinician-Administered PTSD Scale (CAPS-5; Total Severity Score). Second, group (MDMA vs. placebo) differences in methylation change were assessed for sites that predicted treatment response.

**Results:**

Methylation change across groups significantly predicted symptom reduction on 37 of 259 CpG sites tested, with two sites surviving false discovery rate (FDR) correction. Further, the MDMA-treatment group showed more methylation change compared to placebo on one site of the *NR3C1* gene.

**Conclusion:**

The findings of this study suggest that therapy-related PTSD symptom improvements may be related to DNA methylation changes in HPA genes and such changes may be greater in those receiving MDMA-assisted therapy. These findings can be used to generate hypothesis driven analyses for future studies with larger cohorts.

## Background

Recent investigations demonstrate significant benefits of MDMA (3,4-methylenedioxymethamphetamine)- vs. placebo with therapy in the treatment of PTSD. A pooled analysis of six Phase 2 trials demonstrated that 54.2% of persons treated with MDMA-assisted therapy no longer met criteria for PTSD at study end compared to 22.6% in the control group, and gains continued to be sustained over time (1, 2). These results were recapitulated in a more recent randomized, double-blind, placebo-controlled Phase 3 study (3). The placebo with therapy also resulted in a rather high rate of PTSD loss of diagnosis, though far less substantial than that observed in the MDMA-assisted therapy arm; 67% of MDMA participants no longer met criteria for PTSD at study end compared to 32% in the placebo group. These findings raise important questions about the biological changes that occur in association with treatment response after MDMA-assisted therapy. To date, however, there have not been molecular studies examining predictors of successful MDMA-assisted therapy for PTSD.

Epigenetic mechanisms, such as DNA methylation, particularly of certain hypothalamic-pituitary-adrenal (HPA) axis-related genes, have been implicated in mediating adaptations to life conditions and may potentially serve as molecular markers of brain-body health (4–9). Importantly, alterations of the HPA axis were one of the earliest findings in PTSD (10) and have been repeatedly replicated. There is now ample support for the idea that epigenetic alterations underlie neuroendocrine abnormalities in PTSD and may be implicated in conferring risk for PTSD following trauma exposure (11–13). Epigenetic marks on HPA axis genes have been associated with the prediction and successful outcome of psychotherapy in PTSD (14–17). These and other genes were recently confirmed in a second study examining epigenome-wide correlates of successful psychotherapy in PTSD (18). As such, epigenetic alterations on HPA axis genes may be markers, or even predictors, of successful psychotherapy in PTSD. Taken together, HPA axis gene methylation appears to be a promising epigenetic mechanism for treatment response in PTSD, motivating the current study focus.

Using a sub-sample from the recent Phase 3 clinical trial, we conducted a pilot study investigating the methylation of three key PTSD-relevant HPA genes in association with MDMA-assisted therapy treatment response (3); *NR3C1, FKBP5*, and *CRHR1.* The gene *NR3C1* encodes for a glucocorticoid receptor (GR) which plays a role in the HPA negative feedback loop. The *FKBP5* gene encodes a molecular co-chaperone that interacts with cortisol-GR complexes to regulate its downstream transcription-factor activity. The *CRHR1* gene encodes corticotropin-releasing hormone (CRH) receptor 1, which is one of two receptors in this gene family. These genes were chosen based on prior studies demonstrating a change in DNA methylation associating with treatment response in PTSD (14, 15, 17). We hypothesized that (1) symptomatic reduction of PTSD after placebo and MDMA-assisted therapy would be predicted by changes in DNA methylation on *NR3C1, FKBP5*, and *CRHR1*; and (2) MDMA-assisted therapy group would exhibit more methylation change compared to placebo, related to the additional efficacy conferred by MDMA relative to placebo conditions.

## Materials and methods

### Participants

Participants represent a subsample of patients with severe PTSD from a Phase 3 clinical trial (NCT03537014; *N* = 90 treated) who consented to be in the epigenetic sub-study (*N* = 33; MDMA *n* = 16; placebo *n* = 7) (3). For the parent clinical trial, participants were recruited at 15 study sites through print and internet advertisements, referrals from treatment providers, and by word of mouth. Of the 11 sites located in the USA, seven participated in recruiting for the epigenetic study.

Following an initial phone screening for the clinical trial, participants provided written informed consent and underwent further screening assessments for eligibility in the clinical trial. Eligible participants were enrolled in the trial and began psychiatric medication taper if needed, lasting from 0 days (no taper needed) to 103 days. Only participants who enrolled in the study and met criteria for severe PTSD after the taper period and preparatory therapy were offered the opportunity to proceed with the clinical trial and enroll in the present epigenetics sub-study. A total of *N* = 33 participants in the parent clinical trial consented to participate in this epigenetics sub-study; however 10 of the 33 did not provide post-treatment salivary samples due to COVID-19 related interruptions to the study, leaving a total of *N* = 23 (MDMA, *n* = 16; Placebo, *n* = 7) participants with both pre- and post-salivary samples for our analysis.

This study was conducted in accordance with the principles of the Declaration of Helsinki–for full information on the larger study, see here (3). Ethics approval for this epigenetics sub-study was obtained from University of Southern California Institutional Review Board.

### Intervention procedures

For full information on the intervention in the parent clinical trial, see Mitchell et al. (3).

Participants were randomized and allocated 1:1 to either the MDMA-assisted therapy group or the placebo with therapy group. Randomization was stratified by site and occurred following enrollment confirmation. The treatment period consisted of three 8-h experimental sessions of either MDMA-assisted therapy or therapy with inactive placebo control, spaced ∼4 weeks apart. In each experimental session the participants received a single divided dose of 80–180 mg MDMA or placebo. Each experimental session was followed by three 90-min integration sessions that were spaced ∼1 week apart to allow the participant to understand and incorporate their experience. The first integration session always occurred on the morning after the experimental session, and the remaining two integration sessions occurred over the following 3–4 weeks.

A blinded and centralized independent rater pool was used to conduct DSM-5 (The Diagnostic and Statistical Manual of Mental Disorders, Fifth Edition) diagnostic assessments at screening and to administer the primary outcome measure [i.e., Clinician-Administered PTSD Scale (CAPS-5) for DSM-5]. The independent rater measurements were conducted at baseline and ∼3 weeks after each of the first two experimental sessions *via* video interviews. The primary outcome assessment for the clinical trial was the final CAPS-5 conducted ∼8 weeks after the third experimental session (∼18 weeks after the baseline assessment). Saliva samples were collected from participants on days corresponding with the baseline and outcome CAPS-5 assessments to determine epigenetic changes associated with the clinical intervention and primary outcome of PTSD symptoms as measured by CAPS-5.

### DNA methylation

Saliva samples were collected with Oragene-DNA saliva kits (Ottawa, Ontario, Canada) from 33 participants during the baseline visit. Due to COVID-19 related interruptions to the study, 10 participants were unable to provide follow up samples at the final visit, providing a total of 23 participants with both baseline and final saliva samples (MDMA, *n* = 16; Placebo, *n* = 7). DNA was extracted with a DNA Genotek isolation kit (PT-L2P; Ottawa, Ontario, Canada). Sample yield and purity were assessed spectrophotometrically using a NanoDrop ND-1000 (Thermo Scientific, Wilmington, DE, United States). Approximately 500 ng of DNA was treated with sodium bisulfite using the EZ-96 DNA Methylation Kit (Zymo Research, Irvine, CA, United States). DNA methylation was quantified using the Infinium MethylationEPIC BeadChip (IlluminaEPICarray) run on an Illumina iScanSystem (Illumina, San Diego, CA, United States). Raw Intensity Data (IDAT) files were exported for preprocessing in R with the minfi package (19). A filter was applied to remove probes located on the sex chromosomes. Data was subjected to quality control analyses, which included quantile normalization, checking for sex mismatches, and excluding low-intensity samples (*p* < 0.01). All samples passed quality control. Using the minfi package, data were normalized and annotated with Illumina CpG site probe names. Using the R package EpiDISH (Epigenetic Dissection of Intra-Sample Heterogeneity, 3.8) Robust Partial Correlation (RPC) method, the proportion of estimated epithelial cells was used as a covariate in our statistical models. M-values were used for methylation analysis as has been recommended, especially for the homoscedasticity (20).

### Statistical analyses

To ensure generalizability of the results, the epigenetics sub-sample and the parent clinical trial sample were compared on demographic variables and CAPS-5 baseline and outcome scores. Variables were tested for normality using the Shapiro–Wilk’s method. A Student’s *t*-test was used for normally distributed variables, a non-parametric Kruskal–Wallis rank sum test for non-normally distributed variables, and a χ2 test for categorical variables. The same procedures were used to compare the MDMA and placebo groups in the sample.

All sites annotated to the genes of interest on the Illumina Infinium MethylationEPIC BeadChip were analyzed. For all sites of interest, a Student’s *t*-test was used to compare baseline DNA methylation levels between placebo and MDMA groups. All sites significantly different in DNA methylation between groups pre-treatment were removed from further analyses. Multivariate linear regression modeling was conducted to assess if changes in DNA methylation predicted treatment response across groups (hypothesis 1). CpG change scores were used as an independent variable with CAPS-5 change score (final–baseline) as the dependent variable with sex, age, and proportion of estimated epithelial cells as covariates. Because prior associations between methylation of CpG sites on HPA genes with trauma have mixed directions (either hypermethylation, hypomethylation, mixed findings, or differential effects with no direction specified) [see Figure 4 in Watkeys et al. (21)], we allowed for either an increase or decrease in methylation to predict treatment response by calculating absolute value (absΔ) methylation changes scores to test in the same regression models. Because this is a small pilot study, we report both non-corrected and false-discovery rate (FDR < 0.1) corrected results (22–24).

Lastly, to determine if there was a group difference in methylation change, we tested if sites that significantly predicted treatment response across groups (FDR corrected) demonstrated significant group differences in methylation change (hypothesis 2). We compared MDMA and placebo groups on methylation change score with analysis of covariance (ANCOVA) models while controlling for sex, age, and proportion of estimated epithelial cells. Analyses were performed using various packages in R (25).

A note on nomenclature relevant to the following description of CpG genomic locations: CpG sites are often assessed in the CpG-rich islands in and around promotor regions of genes, and the methylation of these CpG sites are generally associated with gene silencing (26). If a CpG site occurs at a distance situated within 2 kb from an island, the location is referred to “north shore” or upstream and “south shore” or downstream from the island; if it occurs at a location within 2–4 kb then it is referred to as “north shelf” or “south shelf” and any CpG site located further than 4 kb from an island are referred to as “open sea” (27). This study assessed all sites annotated to the candidate genes on the Infinium MethylationEPIC BeadChip (IlluminaEPICarray). Beta- and M-values for all three analyzed genes can be found in Supplementary Data Sheets.

## Results

### Epigenetics sub-study participants

**FIGURE 1:**
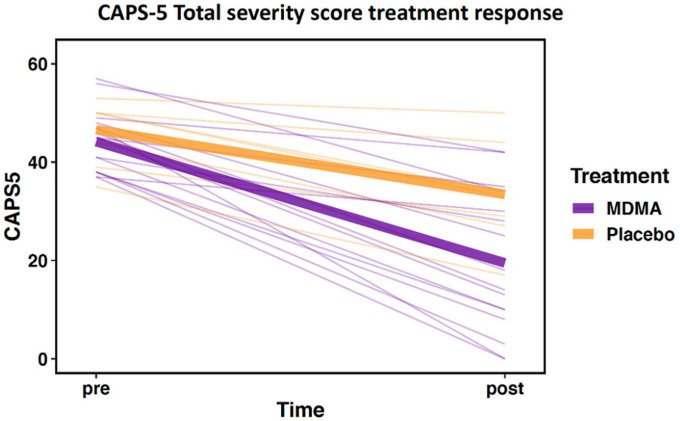
Change in Clinician-Administered PTSD Scale for DSM-5 (CAPS-5) total severity score from pre- to post-treatment. Data demonstrate differential treatment response between 3,4-methylenedioxymethamphetamine (MDMA)-assisted therapy compared to therapy alone (data represent the subset of the clinical trial included in this study; MDMA, *n* = 16; placebo, *n* = 7). Thick lines represent group means, thin lines represent individual scores.

The sub-study sample did not differ from the clinical trial sample in age, sex, baseline CAPS-5, or outcome CAPS-5 scores (Table 1A; all *p*’s > 0.05). Within the sub-study sample, treatment groups (MDMA vs. placebo) did not significantly differ on age or baseline CAPS-5 score but did differ on sex (Table 1B). Sex is used as a covariate in later analyses to account for any bias due to the different group sex compositions. As expected, treatment groups significantly differed on final CAPS-5 score in favor of MDMA conferring more benefit than placebo (Figure 1). A comparison of DNA methylation levels measured from salivary DNA in this study and publicly available methylation level measured from brain can be found in Supplementary Tables 1–3.

**TABLE 1A:** Full clinical study compared to sub-study.

	Full M (SD)/%	Sub. M (SD)/%	Test-value	df	*p*-value	Test performed
Age (years)	41.0 (11.9)	42.05 (12.87)	0.18	1	0.66	Kruskal
Sex (female)	65.60%	61%	0.19	1	0.66	Chi Square
Baseline CAPS-5	44.1 (6.04)	45.16 (6.54)	2.09	1	0.15	Kruskal
Final CAPS-5	25.82 (13.7)	23.32 (14.6)	0.77	62.24	0.44	*t*-test

**TABLE 1B:** Sub-study sample 3,4-methylenedioxymethamphetamine (MDMA) compared to placebo.

	MDMA M (SD)/%	Placebo M (SD)/%	Test-value	df	*p*-value	Test performed
Age (years)	43.4 (13.25)	39.95 (11.91)	0.95	26.98	0.35	*t*-test
Sex (female)	44%	71%	3.58	1	0.05	Chi Square
Baseline CAPS-5	44.2 (6.16)	46.6 (7.03)	−0.97	23.81	0.34	*t*-test
Final CAPS-5	17.94 (14.28)	30.77 (11.02)	−2.82	28.83	0.008	*t*-test

### Relationship between salivary DNA methylation and brain DNA methylation

Beta values (which represent percent methylation at each CpG site) can be visualized for all CpG sites that significantly predicted treatment response (Supplementary Figures 1–3). Correlation between beta values generated from saliva in this study and publicly available beta values generated from brain were compared. *NR3C1* (*r* = 0.73, *p* = < 0.00001), *CRHR1* (*r* = 0.88, *p* = < 0.00001), and *FKBP5* (*r* = 0.83, *p* = < 0.00001) sites all show strong and significant correlations between saliva and brain samples (Supplementary Tables 1–3). Brain DNA methylation values were obtained from the Allen Brain Atlas BrainSpan data.^1^

### Baseline methylation differences between treatment groups

Five *CRHR1* sites, two *FKBP5* sites, and three *NR3C1* sites were significantly different between MDMA and placebo group at baseline thus were removed from further analyses (*p*’s < 0.05).

### Methylation predicting treatment response across groups

#### CRHR1

**FIGURE 2:**
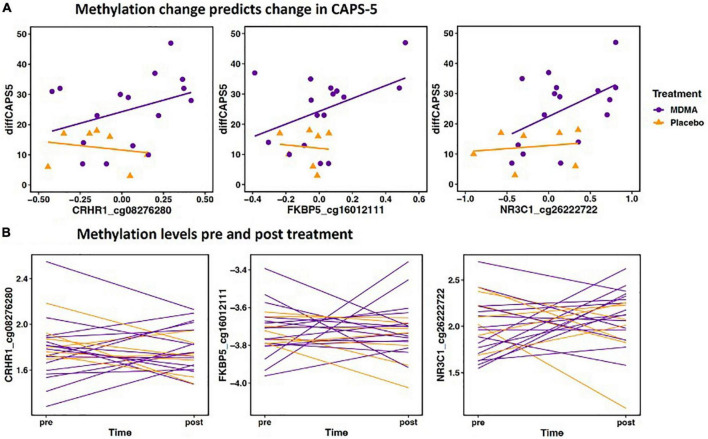
**(A)** Methylation change predicts change in Clinician-Administered PTSD Scale for DSM-5 (CAPS-5). The scatterplot depicts the three Δ change scores with the largest effect size from *CRHR1*, *FKBP5*, and *NR3C1* (x axis) plotted with change scores in PTSD symptoms (CAPS-d). All three sites have a significant positive relationship, meaning decreased methylation is associated with decreased PTSD symptoms. The x axis change scores were calculated as post–treatment mVals–pre-treatment M-vals (positive values represent an increase in methylation). The y axis change scores were calculated as baseline–outcome (positive values represent a decrease in symptom severity). *CRHR1* cg08276280 *B* = 42.12, *p* = 0.005; FKBP5 cg16012111 *B* = 32.52, *p* = 0.012; NR3C1 cg26222722 *B* = 19.32, *p* = 0.003. **(B)** Methylation levels from pre and post treatment (mVals). Figures highlight individual variation in methylation change across groups for the same three sites depicted above. M-values are plotted on the Y- axis; positive M-values represent more molecules are methylated than unmethylated, while negative M-values mean the opposite (20).

Methylation change on 20.9% (17 of 81 sites; 7 Δ; 10 absΔ) of sites annotated to *CRHR1* predicted change in CAPS-5 score (*p*’s < 0.05; Table 2). Ten of these sites resided in open sea regions, two on south shore regions, two on north shore regions, two on an island, and one on a south shelf. The site with the largest Δ effect size survived FDR correction (open sea: cg08276280; *B* = 42.12, *p* = 0.005–pre-FDR correction) and is depicted in Figures 2A, B.

**TABLE 2:** Change in methylation predicting change in Clinician-Administered PTSD Scale for DSM-5 (CAPS-5).

Gene (sites tested)	Relation to Island	Genomic location	Change type (post–pre)	Sig. sites (*p* < 0.05)	*B*	Survive FDR
***CRHR1*** ** (86)**						
cg08276280	os	chr17:43743868	Δ	0.005	42.12	Yes
cg07657976	os	chr17:43887205	Δ	0.006	14.64	∼
cg13947929	ssr	chr17:43863356	Δ	0.016	31.72	∼
cg03423935	nsr	chr17:43697902	Δ	0.026	21.71	∼
cg18090064	os	chr17:43716542	Δ	0.036	12.93	∼
cg08119837	os	chr17:43837500	Δ	0.038	11.87	∼
cg24394631	ssr	chr17:43863000	Δ	0.046	−21.22	∼
cg23420656	ssf	chr17:43865031	absΔ	0.008	29.90	∼
cg14297797	os	chr17:43867801	absΔ	0.011	−17.73	∼
cg22046703	nsr	chr17:43860472	absΔ	0.011	73.01	∼
cg13521908	is	chr17:43861682	absΔ	0.012	−22.14	∼
cg06537391	os	chr17:43799898	absΔ	0.014	38.30	∼
cg05087823	os	chr17:43835721	absΔ	0.021	50.67	∼
cg10106856	os	chr17:43880210	absΔ	0.024	27.08	∼
cg04194664	os	chr17:43716617	absΔ	0.028	−15.52	∼
cg15117716	os	chr17:43871537	absΔ	0.038	−15.71	∼
cg12577105	is	chr17:43860685	absΔ	0.042	19.27	∼
***FKBP5*** ** (59)**						∼
cg16012111	is	chr6:35656758	Δ	0.012	32.52	∼
cg07485685	is	chr6:35696061	Δ	0.024	16.92	∼
cg14339974	os	chr6:35687310	Δ	0.026	−7.23	∼
cg04791658	os	chr6:35611554	Δ	0.035	−31.09	∼
cg07633853	os	chr6:35569471	Δ	0.036	12.64	∼
cg06409316	os	chr6:35642470	Δ	0.039	9.03	∼
cg09318204	os	chr6:35511434	Δ	0.043	20.58	∼
cg08586216	os	chr6:35612351	Δ	0.049	14.08	∼
cg16005389	os	chr6:35592694	absΔ	0.010	23.15	∼
***NR3C1*** ** (114)**						∼
cg26222722	os	chr5:142825390	Δ	0.003	19.32	∼
cg07733851	nsr	chr5:142781498	Δ	0.023	18.93	∼
cg14621978	os	chr5:142735238	Δ	0.027	11.86	∼
cg08423118	os	chr5:142808610	Δ	0.034	−15.39	∼
cg01751279	os	chr5:142793924	Δ	0.052	8.33	∼
**cg01391283**	**os**	**chr5:142907714**	**abs**Δ****	**0.002**	**41.88**	**Yes**
cg03746860	os	chr5:142759375	absΔ	0.009	26.42	∼
cg06770322	os	chr5:142851098	absΔ	0.013	−25.25	∼
cg06613263	nsf	chr5:142779552	absΔ	0.031	−25.46	∼
cg11022710	os	chr5:142820479	absΔ	0.036	37.00	∼
cg11152298	is	chr5:142783383	absΔ	0.047	−49.83	∼
cg01751279	os	chr5:142793924	absΔ	0.047	9.54	∼

os = open sea; is = island; nsr = north shore; ssr = south shore; nsf = north shelf; ssf = south shelf; bold font indicates significantly more methylation change in the MDMA group compared to placebo. A positive direction indicates patients who decrease in symptoms also decrease in methylation; a negative direction indicates patients who decrease in symptoms increase in methylation.

#### FKBP5

Methylation change on 15.8% (9 of 57 sites; 8 Δ; 1 absΔ) of sites annotated to *FKBP5* predicted change in CAPS-5 score [*p*’s < 0.05; Table 2]. Seven of these sites resided in open sea regions and two on an island. No sites from this gene survived FDR correction and the site with the largest Δ effect size for this gene (island site: cg16012111; *B* = 32.52, *p* = 0.012) is depicted in Figures 2A, B.

#### NR3C1

**FIGURE 3:**
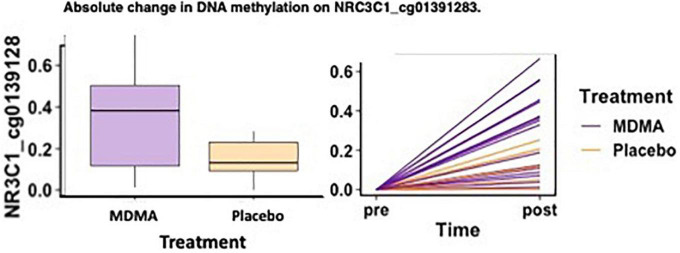
MDMA-assisted therapy group had more absolute change in methylation on NRC3C1_cg01391283 compared to the placebo group. Change in DNA methylation (absΔ) at NRC3C1_cg01391283 from pre-treatment to post-treatment [*F*(1, 18) = 4.78, *p* = 0.042].

Methylation change on 10% (11 of 111 sites; 4 Δ; 6 absΔ; 1 Δ and absΔ) of sites annotated to *NR3C1* predicted change in CAPS-5 score (*p*’s ≤ 0.05; Table 2). Nine of these sites resided in open sea regions, one on north shore regions, one on an island, and one on a north shelf. One site (absΔ; open sea; cg01391283; Figure 3) from this gene survived FDR correction. The site with the largest Δ effect size (open sea: cg6222722; *B* = 19.32, *p* = 0.003) is depicted in Figures 2A, B.

### Group difference in methylation change after treatment

Of the two sites that predicted treatment response across groups after FDR correction (*CRHR1* Δ cg08276280 and *NR3C1* absΔ cg01391283), one site also had a significant difference in methylation change between the MDMA and placebo groups [*NR3C1;* open sea; cg01391283 absΔ; *F*(1, 18) = 4.78, *p* = 0.042]. A figure depicting the absolute change score plotted from pre- to post-treatment can be found in Figure 3.

## Discussion

This is the first study to examine DNA methylation relationships with treatment response to MDMA-assisted therapy in patients with severe PTSD. Due to being a small pilot study, we report both non-corrected significant results and FDR-corrected results. Before FDR correction, seventeen *CRHR1*, nine *FKBP5*, and eleven *NR3C1* CpG sites predicted treatment response across groups. After FDR correction, one *CRHR1* (cg08276280) CpG site and one *NR3C1* (cg01391283) CpG remained significant. The results reinforce previous research that symptom reduction after PTSD treatment correlates with DNA methylation changes on the *CRHR1* and *NR3C1* genes (14, 17). Of the two sites that predicted treatment response across groups after FDR correction, *NR3C1* cg01391283 had a significantly larger change in methylation in the MDMA group compared to placebo.

Genes of the HPA axis have been extensively studied in the context of epigenetic response to trauma (8). The most commonly-studied epigenetic candidate gene in the HPA axis is the glucocorticoid receptor gene (nuclear receptor subfamily 3 group C member 1; *NR3C1*) (21). Glucocorticoid binding at this receptor regulates the stress response through a negative feedback loop (28). Much research to date demonstrates that childhood trauma and early stress alter the methylation status of this gene and its expression [for reviews see Watkeys et al. (21), Jiang et al. (29), and Palma-Gudiel et al. (30)]. While the majority of results find early life stress leads to an increase in methylation and a decrease of expression of *NR3C1*, opposing directional findings are reported (21). In addition to trauma-related methylation changes, two seminal papers found *NR3C1* CpG methylation was associated with PTSD symptoms and many have replicated these findings (12, 31, 32). Taking into consideration the dynamic nature of epigenetic marks and their malleability in response to psycho-social experiences, recent research has focused on potential epigenetic changes in response to psychotherapy for stress-related disorders [for review see here (33)]. Our results add to this growing field, suggesting epigenetic change on the *NR3C1* gene may relate to treatment response to MDMA-assisted therapy. Further, this pilot study was intended to inform hypothesis development, these results suggest specific sites to examine on three HPA genes in future studies with a larger sample size.

The *NR3C1* gene consists of nine non-coding first exons and seven of these are located within a CpG island spanning 3 kb along the proximal promoter region of the *NR3C1* gene (30). The majority of studies assessing *NR3C1* methylation in relation to stress, psychopathology, and treatment response have focused on differential methylation primarily in this CpG island. This work has shaped general discussions about the function of experience-dependent DNA methylation changes. There has been much discussion over the notion of epigenetic modifications turning gene regulation “up or down” or “on or off” since methylation in the immediate vicinity of the transcriptional start site inhibits the initiation of transcription. CpG sites with differential methylation are located all along the *NR3C1* gene sequence and the position of methylation across the gene influences its functionality. For example, methylation in the gene body does not block and might even stimulate transcription elongation or have an impact on splicing (34, 35). While the functional implications of upstream methylation is less understood, some data suggests it may increase expression (36). Indeed, others have found methylation of the *NR3C1* promoter region regulates transcript expression and levels (37–39). In this study, the two sites that predicted change in PTSD symptoms are both in open sea regions. To date, little is known about the functional consequences of DNA methylation in open sea positions. Therefore, it is difficult to speculate on the potential downstream effects of open sea DNA methylation changes on the HPA system.

While the majority of research to date has found increased *NR3C1* promotor methylation associated with trauma, the results are not always consistent. A 2018 systematic review summarized results across studies and effectively visualized how CpG sites in the 1F region of *NR3C1* have been associated with hypermethylation, hypomethylation, non-significant findings, mixed findings, or differential effects with no direction specified, in association with both depression and childhood trauma (21). This suggests that trauma may dysregulate the HPA axis through differing routes, i.e., increase or decrease in methylation at various CpG sites, and that both hyper- and hypo-methylation can be maladaptive (30). Therefore, it stands to reason that psychotherapeutic effects mediating treatment response may occur in opposite directions depending on the individual, gene, and CpG site. For this reason, we chose to not only assess raw difference scores as treatment predictors, but also absolute difference scores which allow for a change in either direction. This novel analytic approach highlighted interesting findings such that the CpG methylation absolute difference score was more sensitive at predicting treatment response than the raw difference scores for *NR3C1* and *CRHR1*. Taken together, these results suggest that both trauma-induced dysregulation and psychotherapy-induced changes of the epigenome may entail either hyper- or hypo-methylation dependent on the individual.

MDMA induces a physiological response similar to an activated stress response, such as increased heart rate, blood pressure, oral temperature, and cortisol levels (40). MDMA and acute stress also increase levels of serotonin, dopamine, and norepinephrine (41–44). While the mechanisms behind acute stress induced changes in DNA methylation are not known, it is plausible that the increased monoamine and cortisol exposure induced by psychological stress may be involved in inducing a transient state of *epigenetic-malleability* (45, 46). Especially taken into consideration that the cortisol-bound glucocorticoid receptor directly up- and downregulates thousands of genes as a transcription factor and other mechanisms (47). Further, the glucocorticoid receptor may directly influence DNA methylation through decreasing activity of a transcription factor, p53, known to regulate DNA methyltransferase (DNMT) (48, 49). However, MDMA-induced psychological effects are quite opposite to an acute stressor or traumatic experience. MDMA produces unique subjective properties such as reduced anxiety, acute positive affect, increased insight, accelerated thinking and euphoria, and increased sense of trust and bonding (50–52). Taken together, MDMA may induce a transient *epigenetic-malleable* state similarly to that of stress but a psychological state highly conducive to successful psychotherapy. Therefore, MDMA-assisted therapy may serve as an “inverse trauma experience,” such that acute stress and MDMA produce similar physiological states and a highly salient psychological experience. However, trauma has the potential to alter epigenetics underlying symptom formation, whereas MDMA-assisted therapy has the potential to alter epigenetics underlying symptom reduction.

While this study highlights potential biological mechanisms underlying MDMA-assisted therapy for PTSD, there are limitations to address. This initial pilot study was small, intended to assess preliminary evidence to support a larger future investigation. As such, additional studies with more power should be conducted to validate these results, however, these results provide specific loci to be studied in the future which may negate the need for *p*-value correction. The small sample size also necessitated the use of a candidate gene approach; while this study focuses on HPA axis genes, there are other potentially informative genes not assessed here [e.g., immune/inflammatory genes such as IL-12 and IFN-γ, for example see Morrison et al. (53)]. However, our results corroborate a large body of literature associating HPA gene methylation and stress-related disorders. While we accounted for participant age, sex, and cell count in our analyses, we were not powered to control for other factors such as race, smoking, ancestry, or trauma histories. Of note, the Infinium EPIC array probes we utilized do not fully incorporate all CpG sites that have been investigated previously. For example, previous studies have focused on the promoter region in intron seven of the *FKBP5* gene, demonstrating epigenetic changes correlating with trauma exposure and PTSD symptomatology (14, 15, 54). However, these sites were not included in the EPIC array so we cannot ascertain if methylation changes in this region were related to MDMA-assisted therapy efficacy. Finally, it is important to note the limitations of using peripheral samples, which cannot elucidate associations in relevant brain tissue.

More than ever, it has become clear that epigenetic changes in response to trauma or stress co-occur with behavioral and physiological symptoms associated with stress-related disorders. Here, we add to a growing number of studies demonstrating that psychotherapeutic experiences may also lead to alterations in the epigenetic landscape underlying symptom improvement. With the small pilot study sample size, it is difficult to determine if the greater efficacy of MDMA-assisted therapy compared to placebo therapy, is driven by greater changes in epigenetic regulation of HPA genes. However, these findings do suggest potential epigenetic mechanisms of MDMA-assisted therapy and their role in symptom reduction is worthy of continued investigation.

## Data availability statement

The original contributions presented in this study are included in the article/Supplementary material, further inquiries can be directed to the corresponding authors.

## Ethics statement

The studies involving human participants were reviewed and approved by the University of Southern California Institutional Review Board. The patients/participants provided their written informed consent to participate in this study.

## Author contributions

BC, JT, DR, BK, CL, RY, and BY-K conceived and planned the experiments. BC organized the data acquisition and IRB approval in collaboration with CH, BY-K, and other MAPS Study Site personnel. BC and CH created the study protocol, instruction manual, trained sites on this sub-study. CL carried out DNA sample preparations, methylation microarrays, conducted all data analyses, and wrote the manuscript. CL and SS processed DNA methylation data. CL and JG created tables and figures. CL, BC, JT, RY, and BY-K contributed to the interpretation of the results. All authors contributed to manuscript revision, read and approved the submitted version.

## Footnotes

1
http://download.alleninstitute.org/brainspan/Methylation/
