# Supplementary figures and images for: Pilot study suggests DNA methylation of the glucocorticoid receptor gene (NR3C1) is associated with MDMA-assisted therapy treatment response for severe PTSD
Source: Front Psychiatry. 2023 Feb 6;14:959590. doi: 10.3389/fpsyt.2023.959590 (PMC9939628; doi:10.3389/fpsyt.2023.959590)

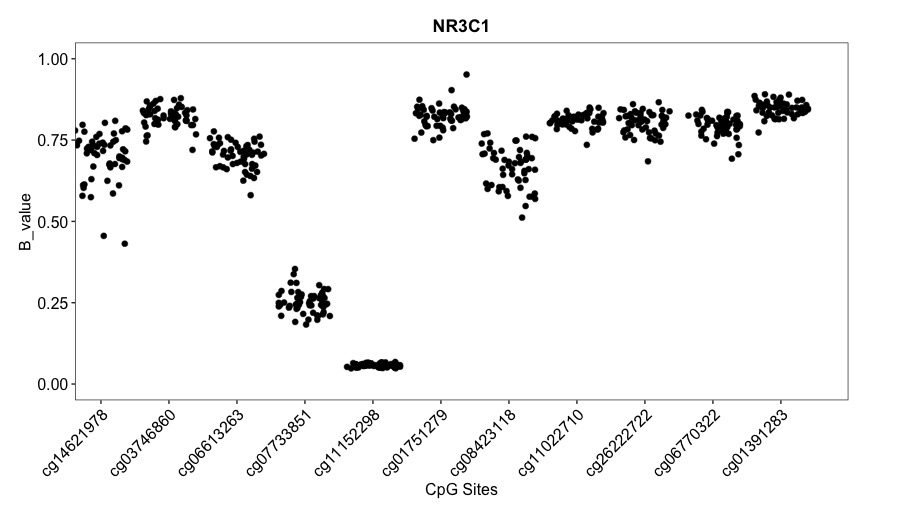

Supplement: Supplementary file 7 [file Image_1.JPEG]

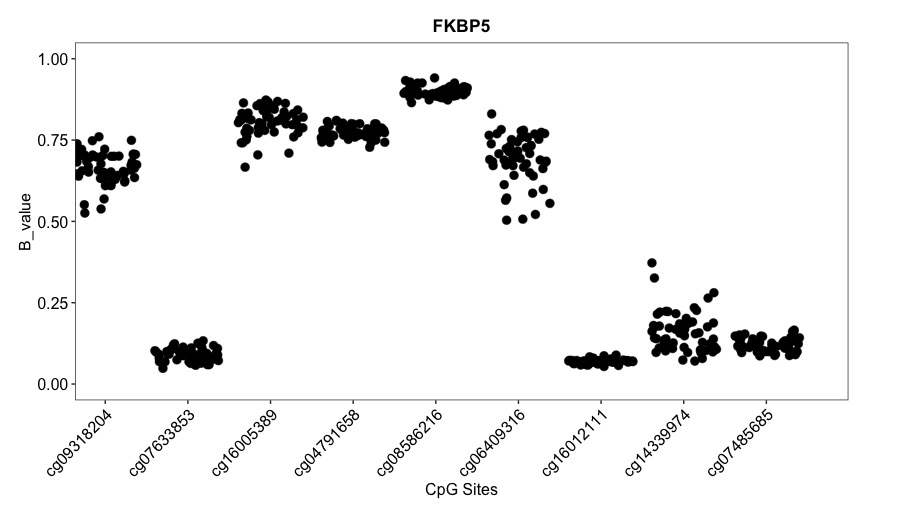

Supplement: Supplementary file 8 [file Image_2.JPEG]

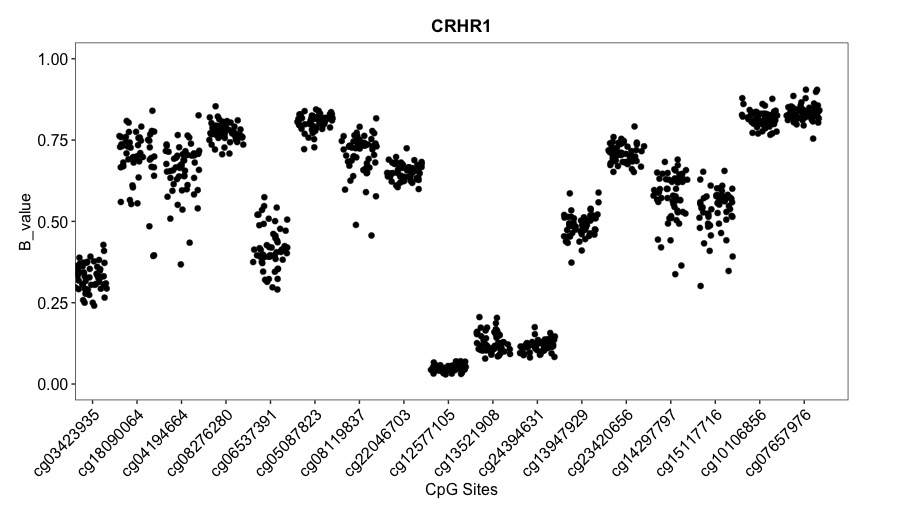

Supplement: Supplementary file 9 [file Image_3.JPEG]
